# β-Carotene prevents bone loss in hind limb unloading mice

**DOI:** 10.3164/jcbn.17-86

**Published:** 2018-05-25

**Authors:** Yu Matsumoto, Yuko Tousen, Yoshiko Ishimi

**Affiliations:** 1 Department of Food Function and Labeling, National Institute of Health and Nutrition, National Institutes of Biomedical Innovation, Health and Nutrition, 1-23-1 Toyama, Shinjuku-ku, Tokyo 162-8636, Japan; 2 Department of Applied Biology and Chemistry, Faculty of Applied Bioscience, Tokyo University of Agriculture, 1-1-1 Sakuragaoka, Setagaya-ku, Tokyo 156-8502, Japan

**Keywords:** hind limb unloading, bone mineral density, β-carotene, osteoprotegerin, calcified bone

## Abstract

β-Carotene has been reported to be useful to maintain a positive balance of bone turnover. However, the effects of β-carotene on bone loss remain to be elucidated in mice with hind limb unloading. Therefore, we investigated whether β-carotene prevented bone loss induced by skeletal hind limb unloading in mice. Female 8-week-old ddY mice were divided into six groups (*n* = 6–8 each) and subjected to: (1) normal housing, (2) sham unloading fed a control diet, (3) hind limb unloading fed a control diet, (4) hind limb unloading fed a 0.025% β-carotene-containing diet, (5) hind limb unloading fed a 0.05% β-carotene-containing diet, and (6) hind limb unloading fed a 0.25% β-carotene-containing diet. After 3 weeks, bone mineral density of the tibia was markedly reduced by unloading, which was prevented by 0.025% β-carotene. Histological analysis revealed a hind limb unloading-induced decrease in the calcified bone of the femur, which was slightly prevented by 0.025% β-carotene. The 0.025% β-carotene-containing diet increased the gene expression of osteoprotegerin in the bone marrow cells in unloading mice. These results suggest that a β-carotene-containing diet may preserve bone health in subjects with disabilities as well as in astronauts.

## Introduction

A lack of weight-bearing physical activity or prolonged exposure to a microgravity environment causes osteoporosis, a common skeletal disease.^(1–3)^ The degree of the reduction in the bone mineral density (BMD) of weight-bearing bone is associated with the degree of immobility and the lack of weight-bearing activity in subjects with disabilities.^(4,5)^ Furthermore, spaceflight induces rapid bone loss, particularly in astronauts exposed to long term spaceflight, who lose an estimated 1–1.6% of BMD per month.^(6,7)^

The tail-suspended hind limb-unloading model is widely accepted as an effective animal model for simulating and investigating weightlessness.^(8)^ In this model, rats or mice are suspended by their tails and placed in a head-down tilt position. Therefore, their hind limbs are subjected to the effects of microgravity. This model results in decreased bone formation and increased bone resorption, and, thus, a concomitant loss of bone mass and a reduction in bone mechanical strength.^(9,10)^

Minerals such as calcium, copper, manganese, and zinc, and vitamins such as vitamin K, and ascorbic acid (vitamin C) are essential for bone health.^(11–14)^ Tang *et al.*^(11)^ have reported that the use of calcium, or calcium in combination with vitamin D supplementation reduces the incidence of osteoporotic fractures in a meta-analysis. Vitamin K is known to play an essential role as a coenzyme in the carboxylation of the γ-glutamic acid protein, and it has been reported that dietary intake of vitamin K has been associated with bone mineral density.^(13)^ Several trace elements, particularly copper, manganese and zinc, are essential for the normal development of the skeleton.^(12)^ On the other hand, it is known that high phosphorus intake acutely and adversely affects bone metabolism in healthy young females.^(15)^ Suzuki *et al.*^(16)^ reported that excessive intake of zinc leads to bone resorption and bone loss in rats.

To prevent the bone loss induced by mechanical unloading, the effects of several osteotropic compounds have been investigated, such as bisphosphonate, 1,25-dihydroxyvitamin D and vitamin K2.^(9,17–19)^ However, because these compounds have associated issues, including side effects, it would be ideal if functional foods were available in addition to a diet. Some studies have reported that food factors prevent bone loss induced by mechanical unloading.^(20–22)^ We recently found that the combination of soy isoflavones and milk basic protein had synergistic effects for the prevention of bone loss in hind limb unloading mice.^(22)^

We also recently demonstrated that β-carotene enhances osteoblastic differentiation and inhibits osteoclast formation *in vitro*.^(23–26)^ β-Carotene is the most abundant provitamin A in the human diet, accumulating in plasma and tissues in the parent compound form. Furthermore, several epidemiological reports have suggested that β-carotene and vitamin A beneficially affect bone health in older adults.^(27–29)^ However, it remains to be elucidated whether β-carotene protects against bone loss in the situation of mechanical unloading.

We hypothesized that β-carotene may regulate bone remodeling and prevent bone loss caused by mechanical unloading. In this study, we investigated the dose-response effects of β-carotene on BMD, bone strength, bone histological parameters and bone metabolism-related gene expression in the bone marrow cells in hind limb unloading mice.

## Materials and Methods

### Animals and experimental design

Seven-week-old female ddY mice (SLC, Shizuoka, Japan) were housed in individual cages in a temperature- and humidity-controlled (23 ± 1°C and 60 ± 5% relative humidity) room with a 12-h light/dark cycle. After 1 week of acclimation, the mice were randomly divided into six body weight-matched groups: normally housed group (Normal, *n* = 6), loading group (Loading, *n* = 6), hind limb unloading group fed a control diet (UL, *n* = 6), hind limb unloading group fed a 0.025% β-carotene-containing diet (UL-0.025% CA, *n* = 8), hind limb unloading group fed a 0.05% β-carotene-containing diet (UL-0.05% CA, *n* = 8), and hind limb unloading group fed a 0.25% β-carotene-containing diet (UL-0.25% CA, *n* = 8). The mice were fed an AIN-93G diet^(30)^ with corn oil instead of soybean oil with or without β-carotene (purity: >97%; Wako, Osaka, Japan). Sodium cholate (purity: >98%; Wako) was added to the diet for 3 weeks to increase the absorption of β-carotene (Table 1). The sodium cholate and β-carotene concentrations in the diets were determined by a previous study.^(31)^

Hind limb unloading groups were subjected to unloading using a tail suspension model.^(8)^ The Loading group was equipped for tail suspension, but the mice were allowed to place their limbs on the cage bottom. The mice were fasted overnight before dissection. The mice were euthanized by exsanguination under anesthesia with pentobarbital sodium (70 mg/kg body weight). Blood was collected by cardiac puncture into a heparin-coated syringe. The plasma was separated by centrifugation at 700 × *g* for 20 min at 4°C and then stored at −80°C until analysis. For BMD measurements, the left tibia was removed and stored in 70% ethanol. The right tibia was removed to extract total RNA from bone marrow cells. For histological analyses, the right femur was removed and stored in 70% ethanol at 4°C. All procedures were performed in accordance with the National Institute of Health and Nutrition Guidelines for the Care and Use of Laboratory Animals in Japan.

### Radiographic analysis of the tibia

The left tibia BMD was measured by dual-energy X-ray absorptiometry (DXA; DCS-600EX-IIIR; Aloka, Tokyo, Japan). The scanned area of each tibia was divided into three equal regions (proximal, midshaft and distal) to assess regional differences.

### Analysis of the strength parameters of tibiae by microcomputed tomography

Proximal tibia were scanned at 48 µm intervals using a LaTheta experimental animal computed tomography system (Model LaTheta LCT-200; Hitachi Aloka Medical, Tokyo, Japan). Proximal tibia analyses were performed in a region of trabecular bone in the epiphysis extending approximately 34 mm towards the diaphysis, excluding the outer cortical bone. The minimum moment of inertia of cross-sectional areas (MMICA) and the polar moment of inertia of cross-sectional areas (PMICA) were calculated using LaTheta software (ver. 1.31; Hitachi Aloka Medical).

### Bone histological stain analysis

Right femurs were embedded in glycolmethacrylate without decalcification. Then, serial sections (3 µm thick) were cut longitudinally using a microtome (model 2255; Leica Microsystems, Wetzlar, Germany). Sections were stained with Villanueva Goldner stain to discriminate between mineralized and unmineralized bone, and identify cellular components. In Villanueva Goldner-stained sections, calcified bone is represented as green and osteoids are represented as red. Histological analyses were conducted by Kureha Special Laboratory (Iwaki, Japan).

### Analysis of plasma albumin concentrations

Serum albumin concentrations were measured with the WAKO A/G B Test (Wako), according to the manufacturer’s instructions.

### RNA extraction and quantitative real-time PCR

 Total RNA was extracted from bone marrow isolated from the right tibia using Isogen II (Nippon Gene, Tokyo, Japan), according to the manufacturer’s protocol. Complementary DNA (cDNA) was synthesized from total RNA using PrimeScript^TM^ RT Master Mix (Takara, Shiga, Japan). cDNA was quantified by real-time reverse transcription polymerase chain reaction (RT-PCR) using the MiniOpticon^TM^ Real-Time PCR System (Bio-Rad, Hercules, CA) and SYBR^®^ Premix Ex Taq^TM^ II (Takara). Cycling conditions were 95°C for 30 s, followed by 40 cycles at 95°C for 5 s and 60°C for 30 s. The primer sequences are shown in Table 2. Results from the bone marrow cells are expressed as the fold change relative to normal mice after normalization to 36B4 gene expression levels.

### Statistical analysis

Data are expressed as means ± standard error of the mean (SEM). The data were assessed by one-way analysis of variance (ANOVA) and the Tukey-Kramer test. Statistical significance was defined as *p*<0.05. Statistical analysis was performed using SPSS ver. 19 (IBM, Armonk, NY). In this study, we did not include the normal group in the statistical analyses.

## Results

### Body weight gain, total food intake, and plasma albumin concentrations

**Fig. 1:**
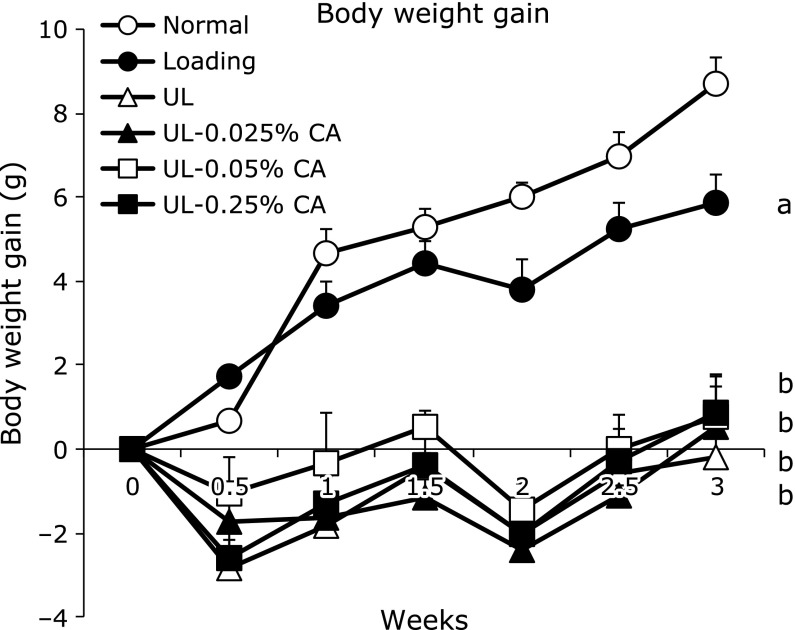
Body weight gain in hind limb unloading mice. Values are expressed as mean ± SEM, *n* = 6–8; means with different letters differ, *p*<0.05. All differences were analyzed by multiple comparison with one-way ANOVA and Tukey-Kramer test. Normally housed group (Normal), sham unloading group fed a control diet (Loading), hind limb unloading group fed a control diet (UL), hind limb unloading group fed a 0.025% β-carotene-containing diet (UL-0.025% CA), hind limb unloading group fed a 0.05% β-carotene-containing diet (UL-0.05% CA), and hind limb unloading group fed a 0.25% β-carotene-containing diet (UL-0.25% CA).

The initial body weight did not differ significantly among the five groups: Normal, 28.5 ± 0.2 g; Loading, 27.2 ± 0.5 g; UL, 27.8 ± 0.7 g; UL-0.025% CA, 27.8 ± 0.4 g; UL-0.05% CA, 27.7 ± 0.5 g; UL-0.25% CA, 27.9 ± 0.3 g. However, the final body weights in UL, UL-0.025% CA, UL-0.05% CA, and UL-0.25% CA groups (23.9 ± 0.3 g, 23.8 ± 0.7 g, 24.8 ± 0.7 g and 24.4 ± 0.6 g, respectively) were lower than those in Normal and Loading groups (34.4 ± 0.8 g and 30.2 ± 0.8 g, respectively). The body weight gains of the mice in UL, UL-0.025% CA, UL-0.05% CA and UL-0.25% CA groups were lower than those in the Loading group (Fig. 1). There were no significant differences in total food intake (Normal, 167.2 ± 2.3 g; Loading, 165.7 ± 3.1 g; UL, 157.6 ± 1.9 g; UL-0.025% CA, 156.6 ± 3.7 g; UL-0.05% CA, 163.3 ± 1.8 g; UL-0.25% CA, 155.3 ± 3.2 g) or plasma albumin concentrations (Normal, 2.61 ± 0.13 g/dl; Loading, 2.29 ± 0.16 g/dl; UL, 2.33 ± 0.11 g/dl; UL-0.025% CA, 2.50 ± 0.08 g/dl; UL-0.05% CA, 2.33 ± 0.07 g/dl; UL-0.25% CA, 2.71 ± 0.03 g/dl) among the five groups.

### BMD and bone strength of the tibia

**Fig. 2:**
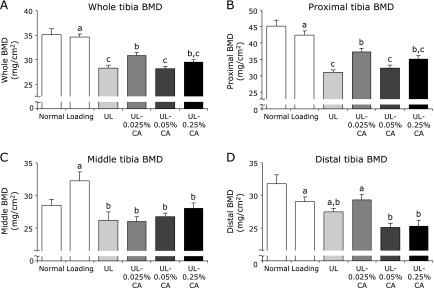
Bone mineral densities in tibia in hind limb unloading mice. Values are expressed as mean ± SEM, *n* = 6–8; means with different letters differ, *p*<0.05. All differences were analyzed by multiple comparison with one-way ANOVA and Tukey-Kramer test. Bone mineral density (BMD) was measured by dual-energy x-ray absorptiometry analysis. (A) Whole tibia BMD. (B) Proximal tiba BMD. (C) Middle tibia BMD. (D) Distal tibia BMD. Normally housed group (Normal), sham unloading group fed a control diet (Loading), hind limb unloading group fed a control diet (UL), hind limb unloading group fed a 0.025% β-carotene-containing diet (UL-0.025% CA), hind limb unloading group fed a 0.05% β-carotene-containing diet (UL-0.05% CA), and hind limb unloading group fed a 0.25% β-carotene-containing diet (UL-0.25% CA).

The BMDs of tibiae in each group are shown in Fig. 2. The DXA analysis indicated that whole and proximal tibia BMDs in the UL group were significantly lower than those in the Loading group (Fig. 2A and B). In contrast, whole and proximal tibia BMDs in the UL-0.025% CA group were significantly higher than those in the UL group (Fig. 2A and B). BMD of the middle region of the tibia in the Loading group was significantly higher than that in the UL group (Fig. 2C). However, there was no significant difference in BMD of the middle tibia among UL and β-carotene treatment groups (Fig. 2C). BMD of the distal tibia in the UL group was slightly lower than that in the Loading group, and the distal tibia BMD in the UL-0.025% CA group was slightly higher than that in the UL group (Fig. 2D). However, the distal tibia BMDs in UL-0.05% CA and UL-0.25% CA groups were slightly lower than those in the UL group (Fig. 2D).

**Fig. 3:**
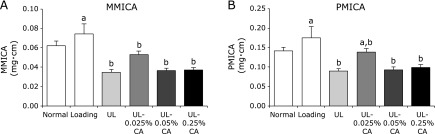
Bone strengths in tibia in hind limb unloading mice. Values are expressed as mean ± SEM, *n* = 6–8; means with different letters differ, *p*<0.05. All differences were analyzed by multiple comparison with one-way ANOVA and Tukey-Kramer test. Bone strength parameters were measured by micro-computed tomography scanning. (A) Minimum moment of inertia of cross-sectional areas (MMICA). (B) Polar moment of inertia of cross-sectional areas (PMICA). Normally housed group (Normal), sham unloading group fed a control diet (Loading), hind limb unloading group fed a control diet (UL), hind limb unloading group fed a 0.025% β-carotene-containing diet (UL-0.025% CA), hind limb unloading group fed a 0.05% β-carotene-containing diet (UL-0.05% CA), and hind limb unloading group fed a 0.25% β-carotene-containing diet (UL-0.25% CA).

To confirm the effect of β-carotene treatment on bone strength of the proximal tibia in unloading mice, bone morphometric analyses were performed by microcomputed tomography. The MMICA and PMICA, which are parameters of bone strength, of the proximal tibia in the UL group were significantly lower than those in the Loading group (Fig. 3A and B). There was no significant difference in the MMICA of the proximal tibia among UL, UL-0.025% CA, UL-0.05% CA and UL-0.25% CA groups (Fig. 3A). Conversely, the PMICA of the proximal tibia in the UL-0.025% CA group tended to be higher than that in the UL group (Fig. 3B).

### Bone histological stain analysis

**Fig. 4:**
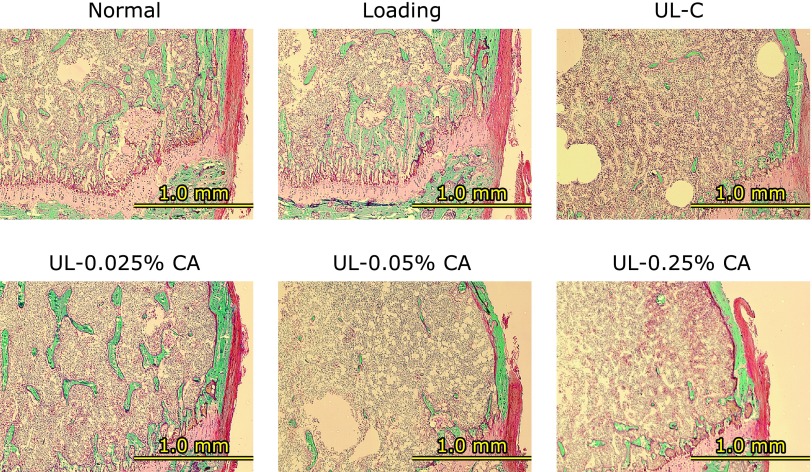
Bone histological stain analysis of distal femur in hind limb unloading mice. The right femurs were fixed, and embedded in glycolmethacrylate without decalcification. Then, serial sections were cut longitudinally, and sections were stained with Villanueva Goldner stain for discrimination between mineralized and unmineralized bone, and for the identification of cellular components. In Villanueva Goldner-stained sections, calcified bone is represented in green and osteoids are represented in red. Normally housed group (Normal), sham unloading group fed a control diet (Loading), hind limb unloading group fed a control diet (UL), hind limb unloading group fed a 0.025% β-carotene-containing diet (UL-0.025% CA), hind limb unloading group fed a 0.05% β-carotene-containing diet (UL-0.05% CA), and hind limb unloading group fed a 0.25% β-carotene-containing diet (UL-0.25% CA).

Histological staining of distal femurs in each group is shown in Fig. 4. Blue-stained areas representing calcified bone in the distal femur were markedly fewer in the UL group than those in the Loading group. The 0.025% β-carotene treatment slightly inhibited this change. There was no difference in the calcified bone in the distal femur among UL, UL-0.05% CA, and UL-0.25% CA groups.

### Quantitation of mRNA expression in bone marrow cells from the tibia

**Fig. 5:**
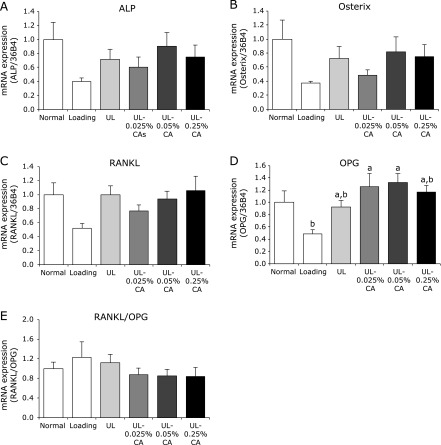
Bone metabolism-related gene expressions in bone marrow cells collected from in hind limb unloading mice. Values are expressed as mean ± SEM, *n* = 6–8; means with different letters differ, *p*<0.05. All differences were analyzed by multiple comparison with one-way ANOVA and Tukey-Kramer test. Expression levels of alkaline phosphatase (ALP), Osterix, receptor activator of NF-κB ligand (RANKL) and osteoprotegerin (OPG) were determined by quantitative real-time PCR. The ordinate axis indicates the relative amount of mRNA compared with Normal mice. Gene expression levels were normalized with 36B4. (A) ALP. (B) Osterix. (C) RANKL. (D) OPG. (E) RANKL/OPG ratio. Normally housed group (Normal), sham unloading group fed a control diet (Loading), hind limb unloading group fed a control diet (UL), hind limb unloading group fed a 0.025% β-carotene-containing diet (UL-0.025% CA), hind limb unloading group fed a 0.05% β-carotene-containing diet (UL-0.05% CA), and hind limb unloading group fed a 0.25% β-carotene-containing diet (UL-0.25% CA).

We investigated the effect of β-carotene on bone metabolism-related gene expression in bone marrow cells collected from hind limb unloading mice. The mRNA expression of alkaline phosphatase (ALP) and osterix in the UL group tended to be higher than that in the Loading group. The 0.025% β-carotene treatment slightly inhibited these changes, but there were no significant differences among the groups (Fig. 5A and B). The mRNA expression of osteoprotegerin (OPG) in UL-0.025% CA and UL-0.05% CA groups was significantly higher than that in the Loading group, and slightly higher than that in the UL group (Fig. 5D). There was no significant difference in the mRNA expression of receptor activator of NF-κB ligand (RANKL) or in the ratio of RANKL/OPG among the groups (Fig. 5C and E).

## Discussion

We investigated the ability of β-carotene to protect against bone loss induced by hind limb unloading. We found that 0.025% β-carotene treatment for 3 weeks prevented the decrease in tibia BMD caused by hind limb unloading. Furthermore, β-carotene treatment increased the mRNA expression of OPG, which regulates osteoclast formation, in bone marrow cells in unloading mice. Several epidemiological reports suggested that β-carotene and vitamin A have beneficial effects on bone health.^(26–29)^ However, the efficacy of β-carotene against bone loss caused by mechanical unloading is not fully understood. Interestingly, although β-carotene has been reported to affect human bone health, there are few animal studies. This is the first report to indicate that β-carotene has beneficial effects on bone health in hind limb unloading mice.

We observed that the decrease in BMD caused by unloading was significantly inhibited by 0.025% β-carotene treatment (Fig. 2A and B), and that 0.025% β-carotene treatment slightly inhibited the reduction in calcified bone caused by unloading (Fig. 4). Some carotenoids, such as β-cryptoxanthin and lycopene, have been suggested to have beneficial effects on bone health in animal studies. Uchiyama *et al.*^(32)^ showed that β-cryptoxanthin prevented estrogen deficiency-induced cancellous and cortical bone loss. Moreover, previous studies have reported that lycopene mainly affects sites enriched in cancellous bone in a postmenopausal osteoporosis model and in normal rats.^(33,34)^ In this study, we observed that 0.025% β-carotene treatment prevented weightlessness-induced bone loss in the proximal tibia that is enriched with cancellous bone (Fig. 2B and 4). Furthermore, bone strength reflects the integration of two main features: BMD and bone quality. Carotenoids, such as β-cryptoxanthin and lycopene, have been suggested to prevent the decrease in bone strength of the femur in estrogen-deficient mice.^(32,33,35)^ Although there were no statistically significant differences in bone strength in our study, 0.025% β-carotene treatment slightly inhibited the reduction in bone strength of the proximal tibia (Fig. 3B). These results suggest that β-carotene might prevent unloading-induced cancellous bone loss with consequent ameliorative effects on bone strength.

Mechanical unloading results in a decrease of bone formation preceded by a transient increase in bone resorption. Therefore, the unloading model reflects imbalanced bone remodeling. In this study, we observed that 0.025% and 0.05% β-carotene treatments increased the mRNA expression of OPG (Fig. 5D). OPG is decoy receptor for RANKL, an osteoclast differentiation factor. Thus, the carotenoid inhibits both the differentiation and function of osteoclasts.^(36)^ Although previous studies have reported that β-carotene affects the expression of various bone metabolism-related genes,^(23–26,37)^ there are no reports on increases in OPG mRNA expression. A recent study reported that β-cryptoxanthin inhibited bone resorption via increases in OPG mRNA expression in human periodontal ligament cells.^(38)^ Thus, similar to β-cryptoxanthin, β-carotene treatment might prevent the bone loss caused by hind limb unloading through the suppression of osteoclast formation via increasing OPG gene expression. Although there were no significant differences among the groups, the mRNA expression of ALP and osterix in the UL group tended to be higher than that in the Loading group (Fig. 5A and B), and 0.025% β-carotene treatment slightly inhibited the increases in expression of ALP and osterix mRNAs that were induced by skeletal unloading (Fig. 5A and B). Bikle *et al.*^(39)^ reported that mechanical unloading increased ALP mRNA expression in bone marrow cells in rats. This result suggests that β-carotene may be regulated by a compensatory response to the decline in osteoblast maturation.

β-Carotene is a major precursor to vitamin A, and it is metabolized to vitamin A by a cleavage enzyme. It is unknown whether the mechanism of β-carotene action in bone metabolism is mediated by provitamin A or vitamin A. Previous reports have shown that retinoic acid, an active form of vitamin A, has a suppressive effect on osteoclast formation and nuclear factor of activated T-cells, cytoplasmic 1 (NFATc1) expression via retinoic acid receptors (RARs).^(5,25,40)^ Recently, we reported that β-carotene might enhance osteoblast differentiation, at least in part via RAR signaling,^(26)^ and that it inhibits osteoclast differentiation of RAW cells by suppressing c-Fos and NFATc1 mRNA expression, which are key factors in osteoclast formation.^(25)^ Thus, we speculate that the inhibition of weightlessness-induced bone loss by β-carotene treatment may be associated with vitamin A and its metabolites. Further research is necessary to determine the mechanism of action of β-carotene on bone metabolism.

In the present study, high doses of 0.05% and 0.25% β-carotene in diets did not affect bone loss (Fig. 2), bone strength (Fig. 3), or calcified bone (Fig. 4) in unloading mice. Umegaki *et al.*^(31)^ reported that the concentration of β-carotene in the blood of mice fed a diet supplemented with 0.05% β-carotene was 0.4 µM. Thus, in this study, the β-carotene concentration in the blood in unloading mice in the UL-0.025% CA group was likely to be less than 0.4 µM. Previous studies have reported that treatment with <0.1 µM β-carotene inhibits osteoclast differentiation and decreases the expression of bone resorption-related genes in cells.^(24,25,37)^ On the other hand, it has been reported that excess dietary β-carotene is cleaved eccentrically to yield β-apocarotenoids that are retinoid receptor antagonists.^(41)^ In our study, the excess β-carotene might have been cleaved to yield β-apocarotenoids and antagonize β-carotene in 0.05% and 0.25% β-carotene treatment in unloading mice. Additionally, many factors, such as endogenous hormones, cytokines, and reactive oxygen species, affect bone metabolism in the bone marrow cells in unloading mice compared with cells *in vitro*. In particular, hind limb unloading induces corticosterone, inflammation, and oxidative stress.^(20,22,42)^ These results suggest that the most suitable level of β-carotene to protect against bone loss caused by mechanical unloading might be <0.025% in the diet in mice.

In conclusion, we found that a 0.025% β-carotene-containing diet attenuates bone loss and increases the expression of OPG in the bone marrow cells in unloading mice. These results suggest that a β-carotene-containing diet may preserve bone health in subjects with disabilities as well as in astronauts.

## Author Contributions

Study concept and design: YI and YT; acquisition of data, analysis, and statistical analysis: YM and YT; interpretation of data: YM, YT and YI; drafting of the manuscript: YM and YT; critical revision of the manuscript for important intellectual content; YI and YT; obtained funding and study supervision: YI.

## Figures and Tables

**Table 1:** Composition of the experimental diets^a^

Ingredient	Control	0.025% β-carotene	0.05% β-carotene	0.25% β-carotene
	g/100 g diet			
Corn starch	52.95	52.9	52.85	52.45
Casein milk	20	20	20	20
Sucrose	10	10	10	10
Corn oil	7	7	7	7
Cellulose	5	5	5	5
Mineral mixture	3.5	3.5	3.5	3.5
Vitamin mixture	1	1	1	1
l-Cystine	0.3	0.3	0.3	0.3
Choline bitartrate	0.25	0.25	0.25	0.25
*tert*-Butylhydroquinone	0.0014	0.0014	0.0014	0.0014
β-carotene	—	0.025	0.05	0.25
Sodium cholate	—	0.025	0.05	0.25

^a^Prepared according to AIN-93G formulation.

**Table 2:** Sequence of primers used for real-time PCR

Gene	Forwad primer (5' to 3')	Reverse primer (5' to 3')
36B4	GGCCCTGCACTCTCGCTTTC	TGCCAGGACGCGCTTGT
Osterix	CCCTTCTCAAGCACCAATGG	AGGGTGGGTAGTCATTTGCATAG
ALP^1^	ACACCTTGACTGTGGTTACTGCTGA	CCTTGTAGCCAGGCCCGTTA
RANKL^2^	TGAAGACACACTACCTGACTCCTG	CCACAATGTGTTGCAGTTCC
OPG^3^	TCCTGGCACCTACCTAAAACAGCA	ACACTGGGCTGCAATACACA

^1^Alkaline phosphatase, ^2^Receptor activator of nuclear factor κB ligand, ^3^Osteoprotegerin.

## Abbreviations

ALP: alkaline phosphatase
ANOVA: one-way analysis of variance
BMD: bone mineral density
DXA: dual-energy X-ray absorptiometry
MMICA: The minimum moment of inertia of cross-sectional areas
NFATc1: nuclear factor of activated T-cells, cytoplasmic 1
OPG: osteoprotegerin
PMICA: the polar moment of inertia of cross-sectional areas
RANKL: receptor activator of NF-κB ligand
RARs: retinoic acid receptors
SEM: standard error of the mean
